# Epidemiology of unintentional injuries among 15–24-year-old vocational school youth from Peshawar Pakistan: A cross-sectional survey

**DOI:** 10.21203/rs.3.rs-2603081/v3

**Published:** 2024-01-31

**Authors:** Sarwat Masud, Adnan A. Hyder, Uzma Rahim Khan, Nadeem Ullah Khan, Ahmed Raheem, Pammla Petrucka

**Affiliations:** Aga Khan University Hospital; George Washington University; Aga Khan University Hospital; Aga Khan University Hospital; Aga Khan University Hospital; University of Saskatchewan

**Keywords:** Adolescents, Burns, Falls, Pakistan, Road Traffic Injuries, Unintentional injuries, Youth

## Abstract

**Background:**

The burden of unintentional injuries among youth (15-24 years) is high. There is paucity of data on unintentional injuries in youth working in Vocational Training Institutes.

**Objective:**

To determine the incidence, characteristics, and risk factors of unintentional injuries among youth.

**Methods:**

**Design:**

**A** retrospective cross-sectional survey was conducted among select vocational school youth in Peshawar, Pakistan between February 2022 to October 2022.

**Participants:**

A total of 547 study participants participated in the survey, 356 were males while 191 were females. Data were collected on using the World Health Organization community survey guide for injuries and violence. Multilevel Negative Binomial Regression model was used to report incidence rate ratios of all unitentional injuries.

**Results:**

**A** total of 503 injuries were reported by the youth, with road traffic injuries being the most common (n=197, 39%), followed by burns (n=89, 18%), falls (n=79, 16%) and poisonings (n=15, 3%), drownings (n=23, 7.1%). Occupational injuries reported during vocational training were (n=95, 18%). Males had a higher incidence rates of RTI 3.24[2.35-5.3], falls 1.30 [0.74-2.27], poisonings 2.14 [0.57-7.58] and drownings 2.46(0.84-7.21), while females had a higher incidence rate of burns 2.19 [1.785-3.46].

Lack of education 4.6 [1.12 −18.91] (*p*=0.034), smoking 1.25 [1.05 −2.69] (*p*=0.049), lack of fathers education 4.71 [2.12 −10.49] (*p*=<0.001), carrying a gun 6.59 [2.54 −17.11] (*p*=<0.001), crowded families 3.59 [3.11 −5.07] (*p*=<0.001), lower family income 2.04 [1.04 −4.02](*p*=0.039*), lack of helmet use 4.54 [2.12 −9.76] (*p*=<0.001) and lack of seat belt use 1.3 [1.14 −1.69] *(p=* <0.001) were significant risk factors for unintentional injuries in youth.

**Conclusion Added value of the study:**

This study is one of the first research studies conducted in vocational school youth in Pakistan. It provides the recent rate of unintentional injuries among the youth of Pakistan. High occupational injuries among vocational school youth were reported which needs further research.

## Introduction

Unintentional injuries UIs are a leading cause of death among youth 10-24 years old [1]. In 2019, 1·49 million youth died (1·39-1·59) globally due to UIs of which 61% were males [2]. Nearly one-third (32·7%) of the above-mentioned deaths were due to transport injuries, unintentional injuries, or interpersonal violence and conflict. Although deaths in this age group have decreased by 30% in females and 15·3% in males since 1950, the sex-based differences in mortality rate has widened in most regions of the world [2]. The World Health Organization confirms road traffic injuries RTI, drowning, and violence as major contributors youth mortality [3]. The Global Burden of Disease (GBD) Report in 2013 identified transport injuries (18·3% [95% UI 16·6–19·7]) as a major cause of death for males between 10 and 24 years of age, followed by self-harm (7·8% [5·9–9·3]); whereas self-harm, RTI, and HIV/AIDS were the leading causes of death for females aged 15–19 years (24·9%), and self-harm, road injuries, and tuberculosis were the leading causes of death for ages 20–24 years (21·9%) [4]. A significant proportion (19%) of the global injury related deaths are occurring in the Eastern Mediterranean Region which includes Pakistan [5] [6].

Pakistan has a population of 216.6 million of which 19.4% are youth aged 15-25 years (12). Pakistan is one of the countries with a high number of youth [7]. The 1^st^ National Injury Survey of Pakistan (NISP), which is based on a survey done in 1997, provides the most recent incidences of unintentional injuries from Pakistan. The incidence of all unintentional injuries according to the NISP in the 16-45 years age bracket, was 45.6 per 1000 per year, while the incidence for road traffic injuries was 15 per 1000 per year (CI: 13.7–16.5). NISP points out the highest incidence of road traffic injuries among laborers at 119.5 per 1000 population, vendors at 106 per 1000 population, businesspersons at 51.9 per 1000, and students at 47.5 per 1000. NISP also identified male sex, age group 16–45 years, and professional laborers/vendors at high risk of road traffic injuries [8]. Another national survey, the 1990-94 National Health Survey of Pakistan (NHSP) [9] reported the annual incidence of all unintentional injuries for the age group from 15-30 years in Pakistan as 46 injuries per 1000 population per year[9]. Furthermore, the annual incidences reported for other causes include: falls at 22.2 per 1000, road traffic injuries at 17 per 1000, poisonings at 3.3 per 1000, and burns at 1.7 per 1000 [9].

Occupational injuries (OIs), although not officially categorized as unintentional injuries, are an important cause of disability and death among youth, as per the Centers for Disease Control and Prevention (CDC) [10]. A higher prevalence of intentional injuries (29%) has been previously reported among youth from vocational schools compared to high school students (17%) [11]. Occupational injuries (also known as work injuries (WIs)), which are injuries that occur at work or in relation to one’s occupation, have been reported to be two times higher among young workers (16-19 years) compared to workers of older ages [12]. Although dedicated studies on OI in vocational youth from Pakistan are rare, one regional study [13] on OIs seen at an emergency department reported higher OIs (47%) among the younger workers (14-18 years) as compared to older workers(18- 35 year) 25% from the Rawalpindi region [13]. The majority experienced hand injuries, with fingers being the most common body part affected (67.5%) while (62.5%) also had fractured bones associated with their hand injuries. Higher OIs (82%) were seen in males compared to females (17%) which is a trend also reflected in international literature [13]

According to UNESCO-UNEVOC approximately one in ten (i.e., 446 000) youth in Pakistan are engaged in secondary vocational learning, the majority being male (67%) [14]. Previous approaches to health surveys of youth have mainly focused on school-based youth as seen in the CDC’s Global School-based Student Surveys (CDC-GSBHS). School-based youth are generally younger (10–17-year age) and does not represent the older youth (18-24 years) that are non-school going. This is a problem, especially in LMICs where a large proportion of youth are not enrolled in schools [4]. Youth at vocational training institutes (VTIs) is a neglected group that is seldom studied [15-18]. We aimed to determine the incidence, characteristics, and risk factors of unintentional injuries among select youth enrolled at the VTIs in Peshawar, Pakistan.

## Methods

### Study design:

This study was part of an explanatory sequential mixed methods study with an overall aim to study unintentional injuries in the youth community in Pakistan [19]. It had two phases denoted as quan → QUAL [19]. Phase 1 comprised of a quantitative (quan) survey, while phase 2 was qualitative (QUAL). In Phase 1 the participants completed a standard World Health Organization questionnaire for injuries and violence in the community [20]. The findings from Phase 1 (quan) are provided in this manuscript.

### Study Setting:

The study was conducted among students of four vocational centers (two male and two female) located in urban areas of Peshawar, Pakistan between February 2022 to October 2022.

### Participants:

Youth at VTIs are a sub-population of youth who are at risk of unintentional injuries. There is a network of four government-run VTIs across Peshawar, Pakistan. These VTIs prepare students for various vocations and skilled jobs. The youth population at VTIs is unexplored in terms of their demographics and behavioral risks for all unintentional injuries.

#### Inclusion criteria

Youth 15-24 years old, enrolled at a VTI in Peshawar (between 2021-22), able to provide written informed consent if 18 years old or written assent and parental consent if less than 18 years. They must be able to read and write Urdu to fill out the questionnaire. (Pashtu is a local spoken language but majority cannot read or write Pashto, 100% of participants read and wrote Urdu).

#### Exclusion criteria:

Any participants absent on the day(s) of the data collection were excluded. (A second attempt was made at recruitment). If a participant or parents requested withdrawal they were excluded.

### Operational definitions:

#### Unintentional injury:

UI was defined as any injury which was unintentional for which medical treatment was received (at a hospital or a clinic or first aid from his/her mates, teachers, or parents) or it was not treated but caused the injured to miss a half day or more of training or regular activities.

The International Classification of Disease ICD-10 classification for unintentional injuries has been used, which includes road traffic injuries, burns, falls, poisonings and drownings [1].

#### Sex:

Sex was defined as male or female based on the biological classification.

### Variables

#### Dependent variable:

##### Total injuries count:

The total injuries count was calculated by adding up the individual injuries counts. Each injury was taken as a mutually exclusive event in previous 12 months. Information was gathered about individual unintentional injuries count including road traffic injuries, falls, burns, drownings, poisonings, in-training injuries, and hand injuries. ICD-10 coding was used for reporting unintentional injuries.

#### Independent variables:

##### Age:

Participants with age 15-24 years were included in the study to prevent confounding due to age.

##### Education:

Information was gathered about mother and father education based on the educational system in Pakistan which included middle school, secondary school, higher secondary school, bachelors, master's and other for uneducated. The categorical variable was then converted into a binary variable as educated and not educated.

##### Family income:

Income information was gathered as per monthly family income and monthly youth income. A composite family income score was generated by adding the two incomes.

### Data Collection Instrument:

The WHO questionnaire for injuries and violence was adapted for this study. The original WHO tool was first published in 2004 as part of guidelines for conducting injury surveys in the community settings [20]. The original WHO questionnaire had 60 questions comprising of sections on sociodemographic, substance use, safety behavior, unintentional injuries, impact of injuries/ disabilities and first aid [20]. The original WHO instrument was designed to gather reliable and valid data in the community setting [20]. Using this instrument on a national sample can provide generalizable and valid results [20]. The WHO instrument was designed by injury experts based on “previous experience” hence the face and content validity this instrument is already established [20]. The reliability of this instrument is unknown [19].

The original WHO questionnaire was modified to include sections about vocational training (Q 4, Q 5, Q 9, Q 12, Q 13) and occupational injuries (Q 55, Q 56, Q 57, Q 58, Q 59, Q 60). Furthermore, questions about counts of RTA, falls, burns, poisonings, and drownings were also included (Q 15, Q 17, Q 15, Q 27, Q 34, Q 42, Q 46, Q 49, Q 52). The modified questionnaire had 81 questions (Appendix A). Pilot testing of the questionnaire was conducted on 50 participants based on which modifications were made to the original WHO tool. The 50 participants from pilot testing were excluded from the final analysis.

### Bias:

The age restriction was done to prevent confounding due to age. Adjustment was done for sex with females taken as reference except for burns where female injuries were reported higher compared to males. Confounding was checked. Restriction of the age group to 15-24 years was done to minimize confounding due to age, and adjustment was also done at analysis stage for age and sex to further control for confounding.

Participants were particularly asked to report counts of their injuries in the last 12 months to prevent recall bias. Verification was done for recall bias on a group of 20 participants who filled the questionnaire twice.

### Study size:

Quantitative sample: 550 (15–24-year-old) youth enrolled at VTIs in Peshawar over the last 12 months were sampled. Assuming an incidence of unintentional injuries among 16-45 years age as 45.6 per 1000 or 0.05% [8] and 3% or 0.03 risk difference of unintentional injuries between males and females [21] while using a two sided test at a confidence level of 95% and a power of 80% a minimum sample of 505 was calculated using the formula n=(Z∝∖2√(Po(1−Po))+Zβ√(Pa(1−Pa)))2∕((Po−Pa)2). Adding a non-response rate of 10% (50) achieved a total sample of 550.

#### Sampling strategy:

During the Phase 1 of this study convenient sampling strategy was used.

### Statistical methods:

Data were cleaned and participants with > 10% missing data were eliminated. The final sample included in the analysis was 547. Descriptive statistics were reported as frequency and percentages. Multivariable Negative Binomial Regression (NBREG) analysis was applied, and adjusted incidence rate ratios were calculated for age and sex. Statistical Analysis Software Stata Corp LLC version 15.1 was used for statistical analysis.

## Results

A total 547 students participated in the study out of 191 (35%) were female and 356(65%) participants were males. The majority of youth were unmarried 502(92%) were educated while a small percentage were uneducated. Maternal education reported by participants was lower at 326(60%), compared to paternal education at 437(80%). A median monthly income of 35000 PKR per family was reported, 239(44%) youth reported they lived in crowded families (>8 family members) as in table 1.

Among the participants there were very few smokers 15 (2.7%), the majority being nonsmokers 532 (97.3%). Substance use was self-reported by 9 (2%) of the participants. A small number of 43 (8%) participants reported that they had carried a gun in the previous 30 days for personal protection. Fifty percent of youth reported the use of a helmet while 240 (43.9%) reported seat belt use as shown in Table 1

Irrespective of sex the RTIs 197(39%) were the most common injuries reported, as shown in table 3. Most RTI incurred were as a driver (26%) as shown in Table 4, while passenger and pedestrian injuries reported were 14.08% and 12.07% respectively (Table 4). RTIs were followed by burns 89(18%) and falls 79(16%) while drownings 28(5%) and poisonings 15(3%) were the least common non-fatal injuries reported by youth (table3). 287(52%) of youth did not receive any first aid services, further details regarding first aid utilization are provided in table 5.

Participants were asked if they had any unintentional injuries in the previous 12 months. The incidence rate reported corresponds to the year 2021-22. Among all unintentional injuries reported the RTIs were the highest 197(39%) (table 3). When comparing male to females, RTIs had the highest incidence rate 3.24[2.35-5.3] in males (table 6). Similarly, males also had a higher incidence of falls 1.30 [0.74-2.27], poisonings 2.14 [0.57-7.58], drownings 2.46(0.84-7.21) and in-training occupational injuries 1.28 [1.19-3.74]. On the contrary, females had the highest incidence rate of burns 2.19 [1.785-3.46] as compared to males (table 6).

The association of sociodemographic risk factors is shown in table 7. It can be noted that the incidence rate of total unintentional injuries was 4.05 times high in males compared to females (<0.001) after adjusting for age. No significant difference was noted in the incidence rate for youth in age ≤ 19 years when compared to youth with age > 19 years. Education of self and father were found to be significant risk factors for unintentional injuries. Youth lacking self-education had an incidence rate of 4.6 [1.12 −18.91] (*p*=0.034) as compared to educated youth. Youth lacking fathers’ education had an incidence rate of 4.71 [2.12 −10.49] (p<0.001) for unintentional injuries compared to youth whose fathers had education. Mothers’ education did not show a significant impact of the unintentional injuries. Youth with low-income status (family income 35000 PKR or less) had 2.04 [1.04 −4.02] (*p*=0.039*) higher incidence of injuries compared to youth with high income status (family income 35000 PKR). Youth that reported carrying a gun for protection when leaving home in the last 30 days had 6.59 [2.54 −17.11] times higher incidence rate (p<0.001). Other significant risk factors noted were lack of helmet use 4.54 [2.12 −9.76] (*p*=<0.001), lack of seat belt use 1.3 [1.14 −1.69] (*p*= <0.001), smoking 1.25 [1.05 −2.69] (*p*=0.049) living in crowded families (family >=8 members) 3.59 [3.11 −5.07] (*p*= <0.001).

## Discussion

In our study we determined the current trends in unintentional injuries among youth from Peshawar Pakistan. This is a first study to our knowledge to determine the in-training occupational injuries along with other unintentional injuries in youth population from Peshawar Pakistan. Our study shows that RTIs remain the top non-fatal unintentional injury in youth irrespective of age and sex. Burns and falls were second and third most common injuries reported after RTIs. Sex was associated with all unintentional injuries, with males having 1-7 times higher incidence of unintentional injuries except for burns which were higher in females compared to males. Occupational injuries were noted to be high. A sex association was noted in occupational injuries as well. The findings of (NHSP 1990-94) [9] a national level survey in Pakistan, shows a high percentage of fall injuries (48%), followed by road traffic injuries 37.6%, while poisonings (7%) and burns (3) were lower in percentages [9]. The current pattern in our study is different from the trend noted in NHSP. Youth reported RTIs were the number one cause of injury in our study while burn and falls were second and third most common cause of injury reported along with occupational injuries. The reason for this shift could be that NHSP survey was done 30 years ago and since then a global shift towards higher RTIs has been noted among youth generally.

The NISP [8] which was another national level injury survey done in Pakistan in 2004 reported a relative risk of RTI as 2.48 times (16–45-year age) and 3.24 in males compared to female, while a relative risk of 1.63 for all other injuries was reported. Our findings are like NISP; our incidence rate of RTIs in males is 3.53 as compared to females which is higher. In a 2021 trauma registry [22] which was made by the Lady Ready Hospital in Peshawar, prospective data were collected in the hospital for a total of 267 trauma patients from Peshawar, it was noted that Motor Vehicle Collison (MVC) was the most common cause of injury 122 (46%). Pedestrians were the most injured (45%), followed by passengers (31%) and drivers (24%). While in our study driver injuries were higher at 48% while passengers (28%) and pedestrians (24%) injuries were lower. This is contrary to the study by Tanoli et [22]. A study published by Shah et al [23] who obtained data from 30 police stations in Peshawar between 2015-16 and analyzed the mode of transport for the RTI, results showed that 74% of fatal and 59% of the non-fatal crashes in Peshawar were pedestrians [23].

Helmet use is a key determinant in decreasing head injuries in RTIs. A study done on crash injuries in children 18 years and younger who rode bicycles and ATVs reported that residence in a county with both lower median income and scholastic graduation rates associated were associated with unhelmeted crashes, and lower median income significantly predicted unhelmeted crashes. This study revealed socioeconomic factors that identify communities with greatest need for injury prevention initiatives [24]. A study done in Bangladesh by Tana et al found a 1.17 times (95% CI 1.02–1.35; p 0.03) higher risk of head injuries in road traffic accidents, in the unhelmeted group compared to the helmeted group [25].

India being a neighboring South Asian country has demographic similarities with Pakistan. An epidemiological survey done on the adolescent population from India, by Reddy et al in 2021 [26], showed that 73% of the adolescents sampled had unintentional injuries [26], this is similar to our study where 503 injuries were reported which is approximately I injury per participant (*N*=547). Reddy et al reported male sex as a statistically insignificant risk factor with OR of 1.0, this is contrary to our study where males had 1.6 times higher incidence for total unintentional injuries, with a statistical significance. Another important risk factor identified by Reddy et al [22] was smoking though insignificant statistically, on the contrary in our study smokers had a 12.8 higher incidence of total unintentional injuries compared to non-smoker (*P* <0.001) when adjusted for age and sex. Other risk factors identified by Reddy et al [22] were overcrowding [26]. This is similar to our study, which showed that youth with > 8 family members had higher incidence of total expected injuries when adjusted for age and sex (*P*=0.002).

The economic situation of a family has shown to impact the injuries, in our study youth with a family income less than 35000 PKR per month had a significantly higher incidence for total unintentional injuries compared to youth with > 35000 PKR family income. In the GBD 2015 study [27] a factor known as “the sociodemographic index” (SDI) was introduced to better understand the sociodemographic risk factors for unintentional injuries. The SDI was created by combining the income, education, and fertility for various countries. A decreasing trend for injuries was noted with increasing SDI which means when the socioeconomic status of the youth increased the burden of injuries decreased. However, it was also reported to be a multifactorial phenomenon [27]. RTIs showed an increasing trend at low and middle SDI but declined at a high SDI. A 10% increase in GDP in a lower-income country (GDP/Capita <1600) is expected to raise the number of road crashes by 7.9%, the number of traffic injuries by 4.7%, and the number of deaths by 3.1% through a mechanism that is independent of population size, vehicle counts, oil use, and roadway availability. Increases in GDP in richer countries appear to reduce the number of traffic deaths, but do not reduce the number of crashes or injuries, all else equal. Greater petrol use and alcohol use are related to more traffic fatalities in rich countries, all else equal [28].

Near drownings reported from India by Reddy et all [26] were 0.6% , falls 36% and burns 19.4%, our findings are different, our percentages for higher burns than falls. Mortality due to unintentional poisoning in LMICs is four times (2/100,000) as compared to higher high-income countries (HICs) (0.5/100,000). Pakistan National Emergency Department Surveillance for unintentional injuries conducted across seven major emergency departments (2010-11) reported a 10% (25/211) incidence of unintentional poisonings among 0–15-year-old children. Males were affected at a higher rate (68%) compared to females (31.8%) [29]. Mortality due to unintentional drowning is the highest among children, especially males in low-SDI to middle-SDI countries. China, India, Pakistan, and Bangladesh accounted for 51.2% of all drowning deaths in 2017 [30]. Our findings confirm a high incidence rate of the drowning of 2.46 times higher in males compared to females.

When compared to the findings from youth in Brazil the prevalence of self-reported occupational accidents was 55% [31]. Data from the New Jersey Department of Education (NJDOE) on vocational youth from December 1998– June 2015 reported that males had (72%) while females had (28%) occupational injuries, these injuries were mild and mostly involved the hand's [32]

## Conclusion

Our study confirms RTI as the common injury among vocational youth followed by burns and falls while poisonings and drownings were the least reported injuries by youth.

Occupational injuries that occurred during the vocational training were high at 95 (18%). Females had a higher incidence rate of burns compared to males, while males had a higher incidence of RTIs, falls, poisonings, and drownings. Continued efforts towards decreasing RTI, burns, falls, and occupational injuries among youth are needed. Youth interventions in Pakistan should focus on youth behavior like carrying a gun, smoking, helmet use, seat belt use, and literacy. Other targeted interventions could focus on families with low socioeconomic status, crowded living situations, and low paternal education. Specific injury prevention programs are needed for preventing injuries in vocational youth.

### Strengths and Limitations:

The major strength of this study is that it reports UIs in the community which are resource-intense and seldom reported. The study is specific to the youth population and results could be generalized. This study reports non-fatal UIs in the community that are often-missed. Occupational injuries in the youth which are often missed have been reported in this study. Data on UIs were gathered as counts over the previous 12 months and reported as IRRs which makes comparisons with other regions and countries easily.

A limited sample was taken which limits the generalizability of its findings. Lack of follow-up of participants, cross-sectional methodology, convenience sampling and fewer female participants were other limitations of this study.

## Figures and Tables

**Table 1:** Demographic, socioeconomic and behavior-related characteristics of youth (N=547) enrolled at vocational training institutes.

Variable	Frequency (percentage) N = 547
**Age group (years)**	
Median (IQR)	19 (18 -20)
Range (Max - Min)	(24 -16)
**Sex**	
Male	356 (54 %)
Female	191 (35 %)
**Marital status of youth**	
Married	45 (8 %)
Unmarried	502 (92%)
**Technical and vocational training institute**	
TVET- Boys A	54 (9.8%)
TVET-Boys B	321 (58.6%)
TVET Girls-A	12 (2.1 %)
TVET Girls B	160 (29.2%)
**Youth education status**	
Non-Educated	12 (2.1%)
Educated	535 (97.8%)
**Mother’s education status**	
Non-Educated	221 (40 %)
Educated	326 (60%)
**Father’s education status**	
Non-Educated	110 (20 %)
Educated	437 (80 %)
**Monthly Income (PKR)**	
Median [IQR]	30000 (40000 - 22000)
**Monthly Income (PKR) Classification**	
<=35000 PKR	272 (50 %)
>35000 PKR	275 (50 %)
**Family Members in Household**	8.4 ±3.8
Range (Max - Min)	(29 -1)
**Family Members Classification**	
<=8 members	308 (56 %)
>8 members	239 (44 %)
**Helmet Use**	
No USE	273 (50 %)
Used	274 (50 %)
**Smoking Status**	
No Smoker	532 (97 %)
Smoker	15 (2.7 %)
**Substance Use**	
No USE	538 (98 %)
Used	9 (2 %)
**Gun Used**	
No USE	504 (92 %)
Used	43 (8 %)
**Seat Belt Use**	
No USE	307 (56.1 %)
Used	240 (43.9 %)

**Table 2:** Types of unintentional injuries along with International Classification of Disease-10 code. Incidence rate ratios of various types of unintentional injuries in youth in the year 2021-22 after adjustment for sex and age.

Types of unintentional injury with ICD-10 code	N=549	Percentage
Road Traffic Injuries (C.1)	197	39
Burns (C.2.3)	135	18
In-training occupational Injuries	95	18
Falls (C.2.1)	79	16
Drowning Category (C.2.2)	28	5
Poisons Category (C.2.4)	15	3
Total injuries count*	549	100
IRR for unintentional injuries	Adjusted IRR [95% Confidence Interval]	
	Male	Age
Total Injuries	1.63 [1.31-2.04]	0.93 [0.88-0.99]
RTI	3.24[2.35-5.3]	0.95 [0.87-1.04]
Poisoning	2.14 [0.57-7.58]	0.85 [0.61-1.16]
Falls	1.30 [0.74-2.27]	0.82 [0.70-0.96]
Burns (female)	2.19 [1.785-3.46]	0.85 [0.77-0.94]
Drowning	2.46(0.84-7.21)	0.89 [0.75-1.05]
Occupational Injuries	1.28 [1.19-3.74]	1.02 [0.89-1.16]

Total injuries count* was created by adding up RTIs, burns, falls, drownings, poisonings, and occupational injuries.

**Table 3:** Role of an injured participant in RTI and type of vehicles causing injury, First aid and healthcare utilization for the unintentional injuries

**Type of vehicle in recent RTI**	
Walking	47 (8.59%)
Nonmotorized vehicle	5 (0.91%)
Bicycle	16 (2.93%)
Motorcycle	164 (29.98%)
Car	33 (6.03%)
Jeep/ van	8 (1.46%)
Bus	4 (1%)
Three-wheel motorized vehicle	6 (1.10%)
Other	264(58%)
**Role in recent RTI**	
Pedestrian	66(12.07%)
Driver	142(26%)
Passenger	77(14.08%)
Other	261(48%)
**Agent in recent RTI**	
Pedestrian	26(4.75%)
Bicycle	12(2.19%)
Motorcycle	70(12.80%)
Motorized vehicle	115(21.02%)
Fixed object	48(8.78%)
Other	276(50%)
**Was First aid received?**	
Yes	188(34%)
No	287(52%)
Other	72(14%)
**Which medical service was availed?**	
Hospital	195(35%)
Clinic	54(10%)
Healthcare center	4(1%)
General Medical Practitioner	23(4%)
Community worker/traditional	5(1%)
Other	266(49%)
**Was admitted to the hospital?**	
No	478(87%)
Yes	68(13%)
**No of days admitted in the hospital**	Mean=0.12, Sd=0.33, range 0-1

**Table 4:** Role of an injured participant in RTI and type of vehicles causing injury.

**Type of vehicle in recent RTI**	
walking	47 (8.59%)
nonmotorized vehicle	5 (0.91%)
bicycle	16 (2.93%)
motorcycle	164 (29.98%)
car	33 (6.03%)
pickup van jeep or minibus vehicle that seats less than 10 people	8 (1.46%)
bus	4 ( 1%)
three-wheel motorized vehicle	6 (1.10%)
Other	264(58%)
**Role in recent RTI**	
pedestrian	66(12.07%)
driver	142(26%)
passenger	77(14.08%)
Other	261(48%)
**Agent in recent RTI**	
pedestrian	26(4.75%)
bicycle	12(2.19%)
motorcycle	70(12.80%)
motorized vehicle	115(21.02%)
fixed object	48(8.78%)
Other	276(50%)

**Table 5:** First aid and healthcare utilization for the unintentional injuries

**Was First aid received?**	
Yes	188(34%)
No	287(52%)
Other	72(14%)
**Which medical service was availed?**	
Hospital	195(35%)
Clinic	54(10%)
Healthcare center	4(1%)
General Medical Practitioner	23(4%)
Community worker/traditional	5(1%)
Other	266(49%)
**Was admitted to the hospital?**	
No	478(87%)
Yes	68(13%)
**No of days admitted in the hospital**	Mean=0.12, Sd=0.33, range 0-1

**Table 6:** Incidence rate ratios of total self-reported unintentional injuries among youth for year 2021-22 with adjustment for sex and age.

Injury types		
	Incidence Rate Ratio IRR [95% Confidence Interval]	
	Male (adjusted)	Age (Adjusted)
Total Injury	1.63 [1.31-2.04]	0.93 [0.88-0.99]
RTI	3.24[2.35-5.3]	0.95 [0.87-1.04]
Poisoning	2.14 [0.57-7.58]	0.85 [0.61-1.16]
Falls	1.30 [0.74-2.27]	0.82 [0.70-0.96]
Burns (female)	2.19 [1.785-3.46]	0.85 [0.77-0.94]
Drowning	2.46(0.84-7.21)	0.89 [0.75-1.05]
Occupational Injuries	1.28 [1.19-3.74]	1.02 [0.89-1.16]

**Table 7:** The association of total injuries count with demographic, socioeconomic and behavioral risk factors in youth.

Factors	Univariate model		Multivariate model	
	IRR [95% CI]	P-value	IRR [95% CI]	P-value
Male gender	3.29 [1.57 −6.92]	0.002*	4.05 [1.84 −8.91]	<0.001*
Age ≤19 years	2.05 [1 −4.2]	0.049*	1.61 [0.8 −3.27]	0.184
Marital Status (Married)	0.6 [0.17 −2.08]	0.421	-	-
Helmet Use (No)	2.12 [1 −4.49]	0.049*	4.54 [2.12 −9.76]	<0.001*
Smoker (yes)	1.29 [1.06 −3.35]	0.023*	1.25 [1.05 −2.69]	0.049*
Gun Use (Yes)	1.16 [1.06 −1.48]	<0.001*	6.59 [2.54 −17.11]	<0.001*
No Seat Belt Used	1.34 [0.6 −0.6]	<0.001*	1.3 [1.14 −1.69]	<0.001*
Self-Education (Non-Educated)	5 [1.15 −21.85]	0.032*	4.6 [1.12 −18.91]	0.034*
Father’s Education (Non-Educated)	3.06 [1.43 −6.55]	0.004*	4.71 [2.12 −10.49]	<0.001*
Mother’s Education (Non-Educated)	0.91 [0.44 −1.86]	0.794	-	-
Family Income (<=35000 PKR)	2.05 [1.01 −4.16]	0.046*	2.04 [1.04 −4.02]	0.039*
Family (>=8 members)	2.37 [1.13 −3.67]	0.009*	3.59 [3.11 −5.07]	<0.001*

Total injuries count* was used as an outcome variable [Total injuries count =RTIs, burns, falls, drownings, poisonings, and occupational injuries].

IRR: Incidence Rate Ratio, (adjusted multivariable IRR)

Negative Binomial Distribution

95% C.I: Confidence interval

## Abbreviations

CDC: Center of Disease and Control
EMR: Eastern Mediterranean Region
AKU-TIRT: Aga Khan University Trauma and Injury Research Training program
OI: Occupational injuries
Stata Corp 15.1: Statistical Analysis Software Stata Corp LLC version 15.1
STROBE: Strengthening the Reporting of Observational studies in Epidemiology
ICD-10: International Statistical Classification of Diseases and Related Health Problems, tenth revision.
UNESCO-UNEVOC: United Nations Educational, Scientific and Cultural Organization, UNEsco and VOCation
UI: Unintentional Injuries
TVET: Technical and Vocational Education and Training
VTI: Vocational Training Institute
YRBSS: Youth Risk Behavior Surveillance System
WHO: World Health Organization
WI: Work Injuries
